# Curation accuracy of model organism databases

**DOI:** 10.1093/database/bau058

**Published:** 2014-06-12

**Authors:** Ingrid M. Keseler, Marek Skrzypek, Deepika Weerasinghe, Albert Y. Chen, Carol Fulcher, Gene-Wei Li, Kimberly C. Lemmer, Katherine M. Mladinich, Edmond D. Chow, Gavin Sherlock, Peter D. Karp

**Affiliations:** ^1^Bioinformatics Research Group, Artificial Intelligence Center, SRI International, CA, USA, ^2^Department of Genetics, Stanford University, CA 94305, USA, ^3^Department of Bacteriology, University of Wisconsin, WI 53706-1521, USA, ^4^Department of Cellular and Molecular Pharmacology, University of California at San Francisco, CA 94158-2140, USA, ^5^DOE Great Lakes Bioenergy Research Center, Wisconsin Energy Institute, WI 53726, USA and ^6^Department of Medical Microbiology and Immunology, University of Wisconsin, WI 53706-1521, USA

## Abstract

Manual extraction of information from the biomedical literature—or biocuration—is the central methodology used to construct many biological databases. For example, the UniProt protein database, the EcoCyc *Escherichia coli* database and the *Candida* Genome Database (CGD) are all based on biocuration. Biological databases are used extensively by life science researchers, as online encyclopedias, as aids in the interpretation of new experimental data and as golden standards for the development of new bioinformatics algorithms. Although manual curation has been assumed to be highly accurate, we are aware of only one previous study of biocuration accuracy. We assessed the accuracy of EcoCyc and CGD by manually selecting curated assertions within randomly chosen EcoCyc and CGD gene pages and by then validating that the data found in the referenced publications supported those assertions. A database assertion is considered to be in error if that assertion could not be found in the publication cited for that assertion. We identified 10 errors in the 633 facts that we validated across the two databases, for an overall error rate of 1.58%, and individual error rates of 1.82% for CGD and 1.40% for EcoCyc. These data suggest that manual curation of the experimental literature by Ph.D-level scientists is highly accurate.

**Database URL:** http://ecocyc.org/, http://www.candidagenome.org//

## Background

Model Organism Databases (MODs) have become tightly woven into the fabric of modern life science research. The creators of MODs such as EcoCyc, *Saccharomyces* Genome Database, Mouse Genome Informatics, *Candida* Genome Database (CGD) and The *Arabidopsis* Information Resource (TAIR) have used manual curation to amass hundreds of thousands of curated assertions (henceforth, *facts*) from >100 000 publications. Ph.D-level biologists read scientific publications, extract key facts from these publications and enter these facts into both structured and unstructured fields in MODs. Manual curation has been widely assumed highly accurate under the idea that PhD-level biocurators can understand and accurately interpret the life science literature, and correctly transcribe the facts that they read. The goal of this work was to assess the accuracy of MOD curation.

MOD accuracy is important because MODs are used daily by thousands of scientists as online encyclopedias and to help interpret new experimental data in the context of existing knowledge. The data captured in MODs are also used to develop gold standards for training and evaluating predictive algorithms in bioinformatics. For example, when developing algorithms for predicting promoters, operons or protein–protein interactions, bioinformaticists use MODs as sources of reference data sets to evaluate and optimize the accuracy of their algorithms (1–3). MODs have received millions of dollars in government funding over the previous 20 years and are widely supported by their respective communities, yet limited data exist regarding their accuracy.

In 2009, the error rate for curated protein–protein interactions in several databases was found to be 3–9% [averaging 3.8% (22/574)] by (4) [after correction of significant analytical errors in the estimates by (5)]. However, Cusick *et al.* and Salwinski *et al.* checked ∼450 protein– protein interaction facts, which is a limited sample from which to infer the accuracy of all biocuration (see Discussion for other related literature). Thus, to determine how well this result generalizes to other types of curated databases, and whether different types of curated data might yield different error rates, more data are required.

To that end, we studied the error rate in biological curation for EcoCyc (6) and CGD (7) as of March 2014, opting to study curation errors where a database assertion is not found in the assertion’s referenced publication, that is, false-positive assertions. (The Discussion section considers other possible types of database errors.) We used a methodology similar to (4, 5) and assessed the accuracy of a limited number of facts from randomly chosen gene pages (the fact set) within EcoCyc and CGD. For each member of the fact set, a validator checked whether the publication(s) cited by the source MOD contained that fact. Thus, our methodology measured the correctness of the facts present within these MODs, but did not assess whether the MODs extracted all facts from each publication, or whether the contents of a MOD reflect the most current state of knowledge.

## Methods

Validation was performed on EcoCyc version 17.5 and on CGD data available in March 2014. Validators (both Ph.D-level scientists and Ph.D students) who were employed by institutions other than the developers of the respective databases performed the fact checking, to reduce potential bias. EcoCyc fact checking was performed by a CGD curator (author MS), by GWL, by KCL and by AYC; CGD fact checking was performed by curators of EcoCyc and MetaCyc (IMK, DW and CF) and by KMM.

The validation procedure was as follows. Each validator was given a target number of 50–200 facts to check, and each performed the following steps until they had checked the target number of facts:

- Consider a gene chosen at random by a web service.
- Choose up to five facts with literature-based support within the gene web page [e.g. ‘enzyme X requires cofactor Y’, ‘enzyme X has K_m_ value Y for substrate Z’ and ‘protein X is annotated with Gene Ontology (GO) term Y’].
- Access the publication cited for that fact and check whether the fact is found within the publication. If found, score ‘yes’ (correct); if not found, score ‘no’ (error).

We used the approach of checking multiple facts per gene for efficiency’s sake, as it allowed a validator to check multiple facts from the same publication, and it decreased the incurred publication-access fees. Because EcoCyc and CGD cover a wide range of types of assertions, a wide range of types of facts were checked, from enzyme activity to transporter activity to protein–protein interactions. Validators were warned of possible sources of confusion, such as different synonyms used to refer to biological entities or different units of measurement used in the databases versus the publications.

We tracked all validation results within a spreadsheet that recorded the following data: the gene, the fact checked for each gene, the status of ‘correct’ versus ‘error’, the publication(s) checked when the status was ‘error’ and a comment by the validator. A database curator then evaluated the results. Validators were informed of the curator’s evaluation and suggested corrections, and given further opportunity to comment.

Software was written to check and remove potential duplications in the randomly chosen gene sets and to tabulate the scoring data.

## Results and discussion

Table 1 summarizes the validation results. Because our study was designed to measure false-positive assertions, the error rate measures precision. Early in the process, we discovered that not only do curators make errors while curating but also validators make mistakes while validating. While checking the errors reported by the validators, we found several cases that we considered validation errors. We also found shortcomings in our validation protocol and instructions. The rigid Yes/No scoring scheme, originally intended to simplify data analysis, did not allow for differentiation based on the type, source and severity of the error. Therefore, we analyzed all reported errors and rescored them where warranted. The Supplementary file contains all scoring data. The column labeled ‘Yes/No’ contains the initial scoring reported by the validators; the column labeled ‘Modify’ contains corrections to the initial scoring that were made by curators with the approval of the validator. All corrected lines in the validation data files are colored red. In Table 1, ‘Facts in error: initial’ reports the number of errors identified by the validators before correction; ‘Facts in error: final’ reports the number of true errors after subsequent analysis.

**Table 1.** Validators = The number of people who performed validation checks on that database

Statistic	EcoCyc	CGD
Validators	4	4
Facts checked	358	275
Facts in error: initial	8	13
Error rate: initial	2.23%	4.72%
Facts in error: final	5	5
Error rate: final	1.40%	1.82%

Lines marked ‘initial’ represent errors initially reported by the validators. Lines marked ‘final’ represent our final adjusted accounting of errors after corrections described in the text.

The types of corrections made to the validation data were as follows. (i) In a few cases, a validator scored a fact as an error (not present in the publication), although the fact was present in the cited publication. (ii) In several cases, validators found facts that indeed were not present in the cited publication, but were correct as evidenced by another publication (often cited in the same gene page). That is, the referenced publication was incorrect or the publication list for a fact was incomplete. Two each of the reported ‘initial’ errors in EcoCyc and CGD were of this type. We considered this type of error to be an error in the metadata (the citation) that would not lead a database user to a false scientific conclusion; therefore, we did not score it as a ‘final’ database error.

Validation and rescoring identified 10 ‘final’ factual errors and an additional 12 errors that were removed from the ‘final’ list of errors for the reasons stated above. The 10 factual errors were of several types. Some free-text statements (such as descriptions of mutant phenotypes) appeared to have been added to the wrong gene/protein in the database. In other cases, GO term assignments were incorrect. For example, the *Escherichia* *coli* ArtQ protein, one of the predicted subunits of an arginine ABC transporter, was annotated with GO:0015426, ‘polar-amino acid-transporting ATPase activity’ in EcoCyc. However, although the function of this protein—as part of a transporter complex—can reasonably be predicted, it is only the entire complex, not the ArtQ subunit alone, that could potentially be annotated with this function. In addition, the curator should not have used an evidence code indicating the presence of experimental data supporting function of the ArtQ subunit in the cited paper. Such curation errors highlight the fact that GO term curation is a complex task requiring significant training.

Several errors appear to have resulted from a curator missing an important piece of information that is not prominently stated in a publication. In one example, enzymatic activities associated with both soluble and membrane fractions are described in detail in one section of the article, whereas peptide sequences that identify the soluble protein Ynk1 are shown in a different section. A curator failed to connect the two facts and assigned an incorrect GO term for cellular component. In another example, a curator missed a figure showing several mutant phenotypes for multiple genes, and instead relied on a confusingly worded summary description of the results in the text, which led to an incorrect phenotype annotation. These examples highlight the need for curators to carefully double check the annotations they make.

Our protocol whereby validators choose the individual facts to check on a gene page might be considered a potential source of bias. In MODs that contain many different types of data, validation results will always depend on the familiarity of the validator with the type of data being validated. For example, a professional biocurator who is familiar with the Gene Ontology may be more likely to validate GO term assignments, and may also be able to identify more errors in GO term assignments. Conversely, a validator who is not familiar with GO may not recognize an erroneous GO term assignment.

As our reported error rates are based on a sampling of facts from the respective databases, we expect fluctuation in the reported error rates each time the validation exercise is repeated. We adopted a bootstrap approach to quantify the uncertainty in our reported error rates. This approach was necessitated by the complexity of our fact-sampling protocol, under which genes are initially selected, and then a (variable) number of facts for each gene are checked. Standard methods to measure variability would have been available under a fact-sampling scheme that selects uniformly at random from all available facts; but such a sampling scheme, although amenable to textbook analysis, requires a prohibitively expensive effort to identify all facts in the database, including those located within free-text (natural language) fields.

In our bootstrap analysis, we created 10 000 artificial samples for each database, using a scheme that mimics as closely as possible our fact-sampling protocol: each bootstrap sample has the same number of facts as the original validation set and consists of genes selected at random with replacement from those seen during validation; each time a gene is selected for membership in the bootstrap sample, all of its validated facts are included (until the sample attains the required size). These 10 000 artificial samples yield a bootstrap distribution of error rates, from which we can calculate measures of variability. For the CGD database, the bootstrap standard deviation, namely 0.78%, furnishes an estimate for the standard error in the reported error rate; a 95% confidence interval for the true database error rate, obtained from the 2.5th and 97.5th percentiles of the bootstrap distribution, is (0.36, 3.64%). For the EcoCyc database, the standard error is estimated at 0.62%, and a 95% confidence interval for the database error rate is (0.28, 2.79%).

## General discussion

We now have a larger data set from which to address the overall accuracy of biocuration. Our study validated a new set of 633 diverse types of MOD facts, uncovering 10 factual errors. The overall error rate of 1.58% found here is similar in magnitude to the 3.8% average error rate in protein–protein interaction data found by Salwinski *et al.*

Clearly, these two studies are not the final word in database or biocuration accuracy assessment. Although other publications have discussed curation errors, they have been anecdotal and have not estimated curation error rates. The potential confusion over whether ‘manual curation’ is used to mean extraction of information from biomedical articles, or whether it is used to mean manual assignment of protein functions via sequence-analysis software—although the two topics are certainly related, as curation of experimentally determined protein functions can be used to supplement and correct functions assigned by sequence-analysis software. Several groups have studied error rates in protein functional annotation (8–10), with (9) concluding that ‘Our results also highlight the value of building and supporting manually curated databases that rely heavily on experimental evidence available from many types of biological experiments’.

Previous discussions of errors in manual extraction of information from publications include discussions of errors that occur in chemical databases and the importance of manual curation and quality-control procedures for ensuring accuracy in such databases (11, 12), and an in-depth analysis (13) of the scientific confusion and curation errors that led to the incorrect attributions of enzyme function in a number of bioinformatics databases. A study of curated protein-interaction data examined variation in curation of the same papers by different database projects, and found that differences in curated facts reflected both divergent curation policies for those databases, as well as curation errors (14). Another study of protein-interaction curation found that improved curation policies and quality-control measures improve curation quality (15), and that consistently following data-dissemination standards is ‘the most effective action a researcher can take to assist database curators and ensure the efficient and accurate deposition of their data into a relevant database’ (16).

The developers of EcoCyc plan to continue assessing its accuracy by using the same methodology used herein. The model of enlisting volunteer students and database users in such an evaluation is a scalable way of assessing database accuracy. A Web site call for validation by users allows accuracy studies to proceed on an ongoing basis, with periodic publication of the results. Providing a more accurate estimate of the database error rate, an ongoing validation process, will lead to a more accurate database because curators will, of course, correct any errors that are identified. We invite interested scientists to contribute to the EcoCyc validation project by following the instructions at http://ecocyc.org/ecocyc-validation-study.shtml.

Our study did not consider several types of database errors. For example, a false-negative error is one where a database curator has curated a publication that contains a fact F, and F is within the curation scope of the database, but F was not included in the database. These errors of omission would be harder to validate, in part because of the difficulty in training validators regarding the curation scope of a given database. Another type of error is an error of specificity, where a publication may demonstrate a fact, but the curator recorded a less-specific version of that fact (for example, annotation to a more general Gene Ontology term). The recorded fact may be correct, but it could have been more specific. Further, the controlled vocabulary to which the gene product is curated can change over time, often with more-specific elements being added, so errors of specificity can accrue over time. Situations may exist where fact F is present in publication P1, but a later series of publications conclusively showed F to be false. If the database contains F with a citation to P1, do we consider the database correct because it correctly captured F from P1, or do we consider the database in error because it does not accurately reflect the modern view of F? Curation of the same type of data across different databases can cause unexpected variability because of differing curation methodology (14, 17).

Clearly manual biocuration is not perfect, and curators can and do make mistakes. However, different MODs use a variety of approaches to ensure or improve the accuracy of their curation efforts. EcoCyc and CGD encourage users to report encountered errors via the ‘Report Errors or Provide Feedback’ link at the bottom of every EcoCyc data page, and via a link labeled ‘Send a message to CGD curators’ on each CGD page. EcoCyc receives approximately one error report every 2 months; CGD receives approximately one to two error reports per year.

In addition, certain systematic methods can be used to identify some types of database errors, although they are unlikely to identify the factual errors identified by validators. For example, EcoCyc has developed 19 different programs to detect a large number of possible errors within the database (in many cases, the errors are repaired automatically). Examples included malformed Enzyme Commission (EC) numbers or nonexistent EC numbers; numeric values that do not satisfy numeric constraints (e.g. a pI value that is not a valid pH; a genome coordinate that exceeds the length of the chromosome); and checks that PubMed references are valid PubMed IDs.

A promising new approach to ensuring MOD quality is generating metabolic models directly from an MOD, and then checking these models for correctness against experimental data sets. EcoCyc has pioneered this approach and used it since 2012 (6, 18), helping to identify incompleteness in both our knowledge and representation of *E. coli* metabolism. The SGD project improves database accuracy by comparing manually curated GO annotations with computationally predicted GO annotations (19). Future efforts to identify potential errors in databases by using automated approaches might include comparing manual GO annotations for orthologous genes in other organisms. Annotations that differ significantly might identify instances that warrant further review.

Consider that ‘database error rate’—meaning the overall error rate observed in a database—may not always be the same as ‘curation error rate’. We would expect the two rates to differ if some of the data within a database were curated and other data were not curated (e.g. high-throughput data loaded from other sources). EcoCyc contains such mixed data (e.g. its data on lethality of gene knockouts are loaded from other sources, and its experimental GO term annotations are added by sources such as EcoliWiki, CACAO and UniProt). ‘Curation error rate’ could alternatively mean the rate of errors in the initial entry of curated information into the database, or the overall error rate in all curated data fields at a given point in time. These two error rates could differ if some of the errors created during initial data entry were later corrected owing to one of the multiple quality-checking procedures just described.

A common criticism of manual curation is that it is both expensive and not scalable to the massive amounts of data generated and published in the biological domain. To what degree could manual curation be supplemented or replaced by automated information extraction (AIE)? The answer clearly depends on the type of data targeted for automated curation. Methods for recognizing the names of biological entities have improved in recent years, with F_1_ scores (a measure of accuracy that combines precision and recall) recently reported to be in the 80–94% range (20). However, unpublished results from the recent BioCreative IV competition show that higher-complexity tasks in automated curation, such as assignment of GO terms from text, is still highly error-prone. Example projects achieved F_1_ scores of 33.9% (http://www.biocreative.org/media/store/files/2013/bc4_v1_20.pdf) or 26% (http://www.biocreative.org/media/store/files/2013/bc4_v1_22.pdf). Thus, the currently available tools are not yet sufficiently reliable to be used in an unsupervised fashion, even for narrow curation tasks.

However, real-world MOD biocurators gather broad types of data, often using reasoning rooted in their experience as biologists, to make annotations and synthesize information. In EcoCyc, curated data on gene function, enzyme activities, metabolic pathways and regulatory networks are stored in >300 different database fields. Currently, no program can accurately extract any one of these information types; extracting all of them accurately is clearly much harder. Biocurators often synthesize information for genes/proteins, metabolic pathways and regulatory interactions, which is a capability that is far beyond AIE. Another set of curator functions that exceeds the capabilities of AIE is tracking inconsistencies and changes in the names of genes, proteins and metabolites, and detecting and resolving disagreements in the literature. It is clear that biocurators accurately perform multiple complex information-extraction tasks that are currently well beyond the capabilities of AIEs.

## Conclusions

We validated the correctness of 633 facts chosen from gene pages in the EcoCyc and CGD databases. Each fact was validated by checking the publication referenced for that fact. Ten factual errors were found, for an overall error rate of 1.58%, and individual error rates of 1.40% for EcoCyc, and 1.82% for CGD. We encourage further studies of this type to gather additional data. We found that involving the MOD user community is a scalable way of performing validation studies.
